# Uncertainty as an operative construct and treatment target in functional neurological disorder

**DOI:** 10.3389/fpsyt.2026.1770123

**Published:** 2026-03-10

**Authors:** Beth K. Rush, Meagan M. Watson, Traci L. Marfilius, Anushka Irani, Brigid Waldron-Perrine, Robin A. Hanks

**Affiliations:** 1 Department of Psychiatry and Psychology, Mayo Clinic, Jacksonville, FL, United States; 2 Department of Neurology, University of Colorado Anschutz Medical Center, Aurora, CO, United States; 3 Department of Social Work, Mayo Clinic, Jacksonville, FL, United States; 4 Department of Rheumatology, Mayo Clinic, Jacksonville, FL, United States; 5 Waldron Neurorehabilitation Psychology, Plymouth, MI, United States; 6 Department of Physical Medicine and Rehabilitation, Wayne State University School of Medicine, Detroit, MI, United States

**Keywords:** clinical translation, functional neurological disorder, intervention, neuroscience, treatment

## Abstract

Functional neurological disorder (FND) is a prevalent neurologic condition, yet existing treatment approaches yield inconsistent outcomes. We propose a unifying framework that conceptualizes FND as a disorder triggered and perpetuated by individuals lacking targeted tools to reconcile the cumulative impact of intolerance of uncertainty (IU) and multidimensional allostatic overload. Expanding upon predictive coding and IU theories from neuroscience and psychology research, we argue that during times of uncertainty, individuals with FND exhibit a higher rate of prediction error but lack the energy, resources, and adaptive capacity to respond. This sustained hyperactivation of the brain and body trigger and perpetuate FND. We propose that FND treatment requires a strategic and progressively tiered behavioral approach. First, the individual with FND must be taught behavioral skills to gain immediate symptom control and shift automatic, hyperactive processes into conscious awareness. Only then, can practices from Acceptance and Commitment Therapy, Dialectical Behavioral Therapy, and Mindfulness Therapy be leveraged. These practices help explicitly identify sources of uncertainty and teach how to effectively respond to hyperactive processes with skills that optimize function, commitment to values, and well-being. This framework offers testable hypotheses and a pathway to more effective, individualized care for FND.

## Introduction

### Global burden of functional neurological disorders

Functional neurological disorder (FND) is a condition characterized by involuntary neurological symptoms that are not localized to a brain, spinal cord, or nerve related injury with diagnostic evaluation (1). FND is heterogeneous and may present with seizure, motor, autonomic, cognitive, and/or sensory symptoms. FND is common, second only to headache, and accounts for 10 to 20% of new outpatient neurology referrals (2). The point prevalence of FND is estimated to exceed that of neurological conditions with centers of excellence for intervention such as Multiple Sclerosis (MS), Amyotrophic Lateral Sclerosis (ALS), and brain cancer (3). Despite this, treatment for FND is scarce or nonexistent in most regions of the world (4–6). People with FND and their families face significant hardship and disability. The economic burden, both personal and institutional, is substantial but likely underestimated due to limited research on the broader impact of FND on family systems and daily functioning (7–9).

### Defining “functional” symptoms

Neurology training and practice is deeply rooted in identifying and localizing the presence of structural or functional abnormalities in the nervous system. When a patient presents with neurologic symptoms, the clinical evaluation is designed to localize symptoms to structural disruption. If exhaustive neurological exams and diagnostic tests fail to reveal a structural cause, the provider often reassures the patient that no identifiable damage was found. In cases of FND, however, this reassurance can be perceived as hollow - patients are still suffering, and providers themselves are often unclear about a path forward.

This diagnostic model, which excels at detecting what is present, struggles when the symptoms originate from an invisible, unlabeled, or immeasurable process. Some symptoms are difficult for patients to articulate or exist below conscious experience. Some tests lack the sensitivity to detect subtle dysfunction, and in some cases, the tools to identify certain problems may not yet exist. These “processes” are still registered and experienced by the brain and body and have negative impact. The absence of a structural explanation can leave patients feeling invalidated, stigmatized, and uncertain about how to manage their symptoms. Clinicians, trained to find concrete evidence to inform management and treatment options, often feel equally at a loss. This ingrained differential and structure-based approach requires recalibration as it leaves clinicians with unintended inflexibility in their investigations and conclusions.

Our current understanding of neuroscience increasingly recognizes functional disruptions in brain networks as legitimate and meaningful, even in the absence of observable anatomical damage. Fatigue and “cognitive fog”, symptoms absent a clear neurological correlate, often persist in MS (10), autoimmune encephalitis (11), brain injury (12) and Alzheimer’s disease and are recognized and treated (13). Persistent symptoms - even when not explained by structural injury - can significantly impair function, quality of life, and participation in daily and community activities. Moreover, functional neurological symptoms can be comorbid in presentation to structural neurological conditions, such as functional seizures with epilepsy, functional dystonia in Parkinson’s diseases, and functional cognitive symptoms following traumatic brain injury (4). In such cases, symptoms may be frequently attributed to psychiatric, sleep-related, or iatrogenic causes. Although these are reasonable clinical considerations, the experience of uncertainty increases when these explanations fail to fully account for the patient’s experience. The patient and provider remain uncertain about what is going on and how best to treat it. Here forward, we define uncertainty as anything that lacks a known, certain outcome. For example, whether a trigger will result in an FND response (i.e., internal uncertainty), or next steps in a treatment pathway (i.e., external or environmental).

Without consensus on language for discussing the influence of currently unmeasurable factors on symptoms, structural and functional neurological disorders are generally considered mutually exclusive, by default, rather than clinical symptomatology/phenotypy characterization. This false dichotomy gets imposed on a decision-tree for treatment, limiting interventions and unintentionally targeting the wrong operative treatment constructs. Consequently, traditional psychological and rehabilitation treatment modalities are “retrofitted” to address functional neurologic symptoms, rather than designed to reflect patients’ lived experiences or a conceptual framework of FND that directly targets meaningful intervention opportunities. Absence of a framework that recognizes both functional and structural aspects of neurological function contributing to the human experience, perpetuates uncertainty for patients, family members, and healthcare providers.

### Challenges in existing interventions: the problem with traditional CBT

Cognitive behavioral therapy (CBT) is one of the most frequently recommended first line interventions for FND (14–17). However, the effectiveness of CBT in reducing the severity or frequency of FND symptoms and symptom impact is inconsistent (16, 17). It remains unclear whether traditional CBT is inherently limited as an intervention for FND by itself, depends on the acumen of the provider, the length of time devoted to treatment, or whether current methods of evaluating treatment outcomes fail to capture its true impact (18).While some individuals benefit from CBT, as demonstrated by some research trials, many do not experience meaningful, sustained, improvement in real world clinical practice (19).

The continued predominant reliance on CBT, a modality that requires cognitive engagement and restructuring, is counterintuitive given that many individuals with FND report subjective cognitive symptoms, regardless of whether objective cognitive impairment is present (20–22). Furthermore, emerging neuroimaging research has revealed distinct patterns of altered brain network activity thought to contribute to this perceived cognitive inefficiency (6, 23). If treatment heavily depends on cognitive strategies, it may not be well suited for a brain with altered network activity reducing an individual’s capacity for effective cognitive engagement, nor for those with perceived deficits in this area (i.e. learned helplessness). Further, it is possible that even CBT-informed treatment lacks a preceding step of intervention that may make the CBT component of the approach more successful. To our knowledge, this has never been directly questioned or explored. This would not be psychoeducation about diagnosis alone to reduce uncertainty, sometimes therapeutic on its own, but rather a skill-based intervention that interrupts the impact of uncertainty that then facilitates CBT effectiveness (24).

### Challenges in existing interventions: traditional rehabilitative therapies alone

Alongside CBT, physical, occupational, and speech therapies are commonly recommended first-line treatment approaches for FND. Some programs incorporate psychological techniques like deep breathing or distraction/re-direction of attention, but the primary focus remains on physical rehabilitation (25). The Physio4FMD trial is one of the most notable studies to examine the clinical effectiveness of specialized motor rehabilitation for functional motor disorder (26). Although self-ratings of motor symptoms and mental health were improved after intensive motor rehabilitation, there was no improvement in self-reported global physical functioning at 12 months post-intervention (27).

Regardless of the specific therapeutic target, mixed treatment outcomes are further complicated by research suggesting that symptoms may remit only to reappear elsewhere in the body, often presenting as a different FND phenotype (4). This phenomenon is referred to as ‘symptom migration.’ To date, there is limited research documenting the frequency of symptom migration in FND, but providers working with individuals of mixed FND phenotype anecdotally comment on this phenomenon and further research is needed. Accordingly, treatments that focus solely on physical rehabilitation targets, or rely heavily on cognitive strategies, have come under increased scrutiny as to whether they can be individually effective as independent treatment interventions or when delivered simultaneously. It is unclear if select interventions for FND target a construct operative to the extent that it can address current FND symptoms *and* prevent symptom migration. Furthermore, there is limited evidence to determine whether all FND symptoms respond to the same treatments, whether individual or group formats are more effective, or which variables define treatment success. The growing number of clinical trials and intervention programs is encouraging (28), though the ambiguity surrounding best intervention approaches and outcomes is felt deeply by patients, families and clinicians (29, 30). Many of the current mechanistic theories of FND emphasize the need to identify and address sources of dysfunction within the individual (i.e., motor weakness, precision-weighting, belief updating, addressing cognitive distortion), however, no *a priori* single treatment focus statistically proves positive treatment outcomes. Of interest, on retrospective analysis of the Physio4FMD trial data, the team found that only greater perception of control predicted clinical global improvement (31).

In efforts to identify consistent themes across patient experiences and clinical observations, one construct consistently emerges: uncertainty. Uncertainty permeates every level of FND lived experience, symptom and symptom impact, clinical care, research, and education about FND yet first line treatment approaches do not directly address skills for managing uncertainty. It is very hard to recognize and/or measure uncertainty and how the brain and body register its effects. That is because uncertainty is not just felt physically, cognitively, or emotionally, but also viscerally. Uncertainty seems to create a palpable lack of safety to the brain. Existing FND treatments rehabilitate aspects of FND, focusing on addressing perceived sources of dysfunction but do not introduce specific behavioral and lifestyle tools that people with FND can use to actively identify and respond to omnipresent uncertainty, thereby improving safety and restoring adaptive capacity to the brain and body. We propose that people with FND build from a foundation of explicit tools to interrupt FND symptoms and to prevent symptoms from migrating from one FND phenotype to another phenotype.

In this way, we believe that tolerance of uncertainty is a transdiagnostic construct in FND that deserves further exploration. We lay out cumulative neuroscience evidence of how uncertainty operates in the brain and nervous system and how this shifts the normal, safe, reliable, and consistent activation pattern into a state of hyperactivation that the brain and body can no longer sustain. We discuss the construct of uncertainty as it relates to healthy body function, the onset of functional neurological symptoms and the perpetuation of FND. We then highlight a strategic treatment approach for FND which involves a translation of neuroscience principles involved in the registration and the creation of adaptive capacity into clinical neuropsychological intervention for individuals with FND.

## Predictive error, uncertainty and FND

Mechanistic models conceptualizing FND are important for considering treatment targets and a stated priority within the FND research agenda (32). Across neuroscience, predictive coding frameworks have rose in prominence over the last 15 years (33), but many clinicians remain unfamiliar with them and thus, translation to clinical medicine and rehabilitation has been slow (34, 35). Understanding the application of predictive coding theory to neurological, psychological, and physical disorders is relevant as it proposes the mechanisms and cascade of events by which symptoms are explained by dysfunction in and between brain networks rather than a sole localized structure (36).

As such, predictive coding theory is increasingly recognized as a probable mechanism for the development and perpetuation of FND - best understood as a brain network disorder (1, 37, 38). The primary assertion of predictive coding theory is that the brain’s primary role is to be an active machine of inference in service of maintaining homeostasis. Distinct from the hierarchical feedforward model of perception, where filtering and translation of sensory signals results in higher-order perception, enhanced theories suggest that perception arises from predictive coding (32). The brain is constantly generating “top-down” predictions about future experiences, and comparing them with a combination of incoming exteroceptive (e.g. sight) or interoceptive (e.g. heart rate) sensory information. Whether conscious or subconscious, mismatch between predictions and sensory data, is the “prediction error”. Predictive coding theories of FND suggest that the brain overemphasizes prediction errors, most commonly due to past experiences or sensory input overload, effectively “overriding” the brain’s ability to make an accurate prediction of future events. Failure to accurately match prediction with sensory input in FND, produces symptoms such as paroxysmal seizures, interruptions of cognition, involuntary movements or autonomic disturbances, among other manifestations (37). In order to treat FND symptoms, it is suggested that treatments must retrain the brain to form accurate predictions, and minimize prediction errors. This cascade is thought to ultimately reduce FND symptoms (1, 37) suggesting that the prediction error is the “pathological” mechanism of FND. We find an exception to this point, noting that prediction error is not exclusive to FND pathology states but regularly occurs in all brains - healthy, structurally compromised or functionally compromised.

## Impact of prediction error in healthy, structurally compromised, and FND neurological models

In healthy brains, prediction errors of the visual system (and other sensory systems) have been studied for decades (39). For instance, perception of a squirrel near trees on a sunny day may actually be sunlight refracting off mulch. If the brain can return to its initial capture of sensory data (“this is a squirrel”), and recalibrate the sensory data present based on recaptured perception and its knowledge of light properties, it reconciles the first prediction as “error,” and makes an alternative and more accurate prediction (“this is light refracting off mulch”) to replace it. The healthy organism, without structural neurological injury/dysfunction, is capable of reprocessing sensory data and updating internal models of what the sensory data means. This alters patterns of activations in the mind and body demonstrating that individuals can learn and master strategies for resetting and rewiring the flow of information. Stated differently, the ability to reconcile prediction error is a renewable resource for the healthy brain because healthy structure guarantees a path to certainty, thereby guaranteeing a path to reconciling error.

In contrast, when prediction error occurs in the context of structural neurological disease or dysfunction, it cannot be updated or resolved because the brain structures required for sensory reintegration and/or association are compromised. This leads to uncertainty for the brain and nervous system. Examples include rapid forgetting as an early-stage symptom of classical Alzheimer’s dementia (40)and visual misperception as an early-stage symptom of diffuse Lewy body disease (37). If assumptions of predictive coding theory are correct, in both disease states, the brain is making a prediction based on the sensory information and accessed associations it has stored to make sense of that information. Put simply, the brain’s first prediction is not always the most accurate prediction. The question of whether the prediction error can be resolved is determined by whether the individual has any strategies for interrupting or reframing the error encountered. In absence of structural system integrity to resolve the prediction error, interventions for adjusting to neurodegenerative disease symptoms require recruitment of external support to reinstate certainty lost by disease or injury. For example, a caregiver may remind a person with rapid forgetting or reassure a person with visual misperception that there is nothing alarming present. In this way, the caregiver, instead of the brain, initiates reconciliation of prediction error for the patient, allowing the brain and body to regain certainty. The patient may not accept this external support, however, this scaffolding may represent the only chance the patient has to reconcile error and help keep the person to keep functioning.

**Figure 1:**
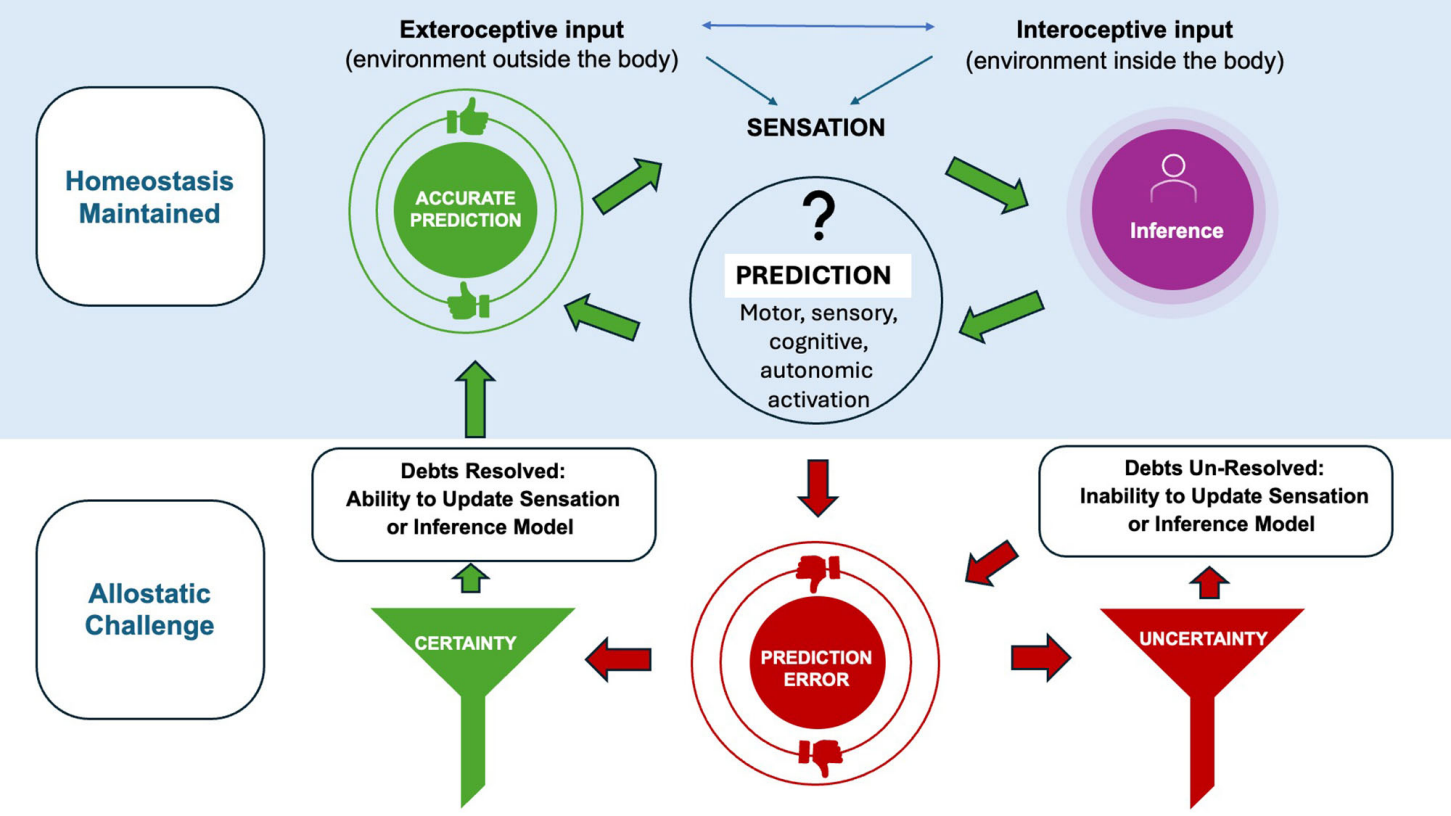
Uncertainty as an operative factor in FND. In healthy states, humans experience sensation which leads the brain to infer what to do next. This inference process automatically triggers a prediction that activates a motor plan, a sensation, an autonomic activation, a feeling or a cognition. This healthy process keeps the brain-body budget running and the organism living. For example, a person feels sweat, the brain infers that it is hot and the person could be at risk of losing too much water so it forces a prediction which may be a chill sensation which stops the person from sweating. This model is not always perfect and sometimes prediction errors occur. If the brain is certain and there is not too much physiological, emotional, cognitive or environmental challenge, this is normal allostasis. The brain can reallocate resources and recalibrate, intentionally re-feeling the sensation of sweat or reconsidering the “automatic” inference that the body is losing water. Making this conscious and certain correction, allows the brain to resolve any potential debt from allostatic challenge, and return to the homeostatic function of cooling the body in response. In FND we propose that prediction errors cannot be challenged due to the fact that allostatic challenge has led to allostatic overload. Allostatic overload, prevents the brain from consciously slowing and updating either sensory registration data or inference model. The prediction error exists in the context of too much physiological, emotional, cognitive or environmental challenge. Therefore prediction error becomes perpetuated, unopposed and unable to return the brain and body to homeostasis until more certainty can be created. Using our example, the brain predicts the body is going to lose too much water and this inference and sensation go un-opposed, so the brain continues the wrong cycle, continuing to cause sweating and perhaps causing additional symptoms of dehydration, pre-syncope or cognitive disturbance until the brain-body budget can be rebalanced.

In FND, a functionally compromised brain, we suggest that the pathology associated with predictive coding errors is not the errors themselves, but rather the process(es) or lack of skills that either limit the brain from updating sensory registration of information, or from updating the internal reference point for making sense of sensory information (**Figure 1**). It is clear that the brain’s first prediction, made off of available sensory data, is not always the most accurate prediction which means the consequent activation of mind and body may not be accurate. In the absence of structural injury, the person with FND should be able to resolve prediction error without external support, returning to homeostatic function, even during transient times of uncertainty, but this likely requires overriding physical, emotional and cognitive valence of the prediction error. We assert that the reconciliation of prediction error can be a renewable resource for the person with functional neurological symptoms, whereas the same internal and personal opportunity is not present for the person with structural neurological challenge. For the latter, adaptive capacity is likely supplied by a source external to oneself, such as a caregiver, or a safe environment. The healthy brain of that support anchors the affected individual in certainty that can no longer be provided to self. Individuals with FND have a healthy brain that can provide certainty to self but they still require anchors of reassurance from a treatment approach that reflects the same safety that the caregiver aids the individual with a structurally compromised brain.

Prediction errors are more likely to occur in the presence of uncertainty regardless of whether the uncertainty is transient or sustained (41, 42). Brain imaging studies demonstrate a peak in neural responses when registering prediction error during high uncertainty, likely reflecting the increased energy it takes the brain to check internal models and adaptively learn to respond to unpredictable sensory input (43). This increases the odds of prediction error but also the likelihood that the individual with the challenges, has less energy and adaptive capacity to reconcile errors that arise. Directly stated, uncertainty complicates prediction and sustains prediction error. Yet uncertainty can never be totally avoided in human experience. Although adverse life events and/or trauma are often thought of as common risk factors for FND, they are no longer recognized as a prerequisite for the development of FND (44). It is possible that simply the inability to tolerate and/or resolve uncertainty, or the lack of learned tools for addressing uncertainty, predisposes someone to prediction error that cannot be resolved.

Rush and colleagues tested this theory by measuring Intolerance of Uncertainty (IU) in a cohort of 45 individuals, with diverse presentations of functional motor disorder (45). Intolerance of Uncertainty (IU) is “a dispositional characteristic that results from a set of negative beliefs about uncertainty and its implications and involves the tendency to react negatively on an emotional, cognitive, and behavioral level to certain situations and events” (46). IU is generally considered a trait that takes longer-term therapy to attenuate, because it is a consistent tendency, however, IU can fluctuate depending on circumstances, making it more state-like at times. In this way, IU may respond to not just long-term cognitive interventions focused on mediating the trait but also having access to immediate coping strategies to reduce IU in an acute state (47). Participants completed an intensive occupational therapy and physical therapy intervention for symptoms over the course of one week with no psychological intervention. Two-thirds of the cohort improved IU and the degree to which IU improved was associated with the degree of improved physical function and activity engagement. This was the first study to formally examine whether IU was a relevant construct in any type of FND and FND intervention. Results suggested that IU was a relevant transdiagnostic construct in heterogeneous presentation of FND and an important variable to consider in treatment.

## Allostatic load, uncertainty and FND

If uncertainty and the ability to respond to uncertainty are operative constructs in FND, then we propose that allostatic load is the threshold or limiting factor of the brain’s capacity to tolerate and reconcile uncertainty. Allostatic load refers to the cumulative physiological effects of exposure to challenges and stressors on the body (48). We are not the first to propose the involvement of allostatic dynamics in the onset and perpetuation of FND. Increasingly, allostatic-interoceptive network (function and dysfunction) schemas offer legitimization to the very functional neurological symptoms that were previously difficult to describe and measure in neurological and psychiatric conditions (49). Historically, allostatic load has been considered from the system-level perspective of biopsychosocial determinants or challenges - such as housing, food insecurity or inequitable access to education. In the context of FND, we ascribe to a more granular perspective via the effect of these biopsychosocial determinants on the brain-body budget. Similar to prediction error, reconciling an allostatic challenge is a normal process of healthy brain-body operation. Increasingly, sources of “allostatic overload” are recognized in relation to physical and mental constructs of illness and disease, and most recent research recognizes allostasis as a core brain function essential for running the budget of the body (49–53).

Allostasis is distinct from homeostasis in that it is the process by which the brain and body energetically adapt to stressors and changes in the environment. Instead of restoring a fixed set point, allostasis modifies the set point based on context (i.e. presence of housing, food, or education). When allostatic load is high, the brain is evolutionarily designed to be focused on addressing the immediate perceived need, and its ability to flexibly respond to uncertainty is reduced, leading to rigid predictions, greater rate of prediction error, poor error correction and difficulty updating beliefs or behaviors. In FND, an abundance of literature suggests that when compared to other debilitating neurological conditions, individuals more commonly experience severe adverse life experiences and/or live in environments with chronic, often uncontrollable stressors (54). For example, we know that a high proportion of individuals with FND are unemployed, on disability or rely on public insurance such as Medicaid (55). Unpredictability, via allostatic challenge, exists as a constant state, rather than a trait defining their lived experience with potential to perpetuate FND symptoms.

## Targeting uncertainty in treatment

**Figure 2:**
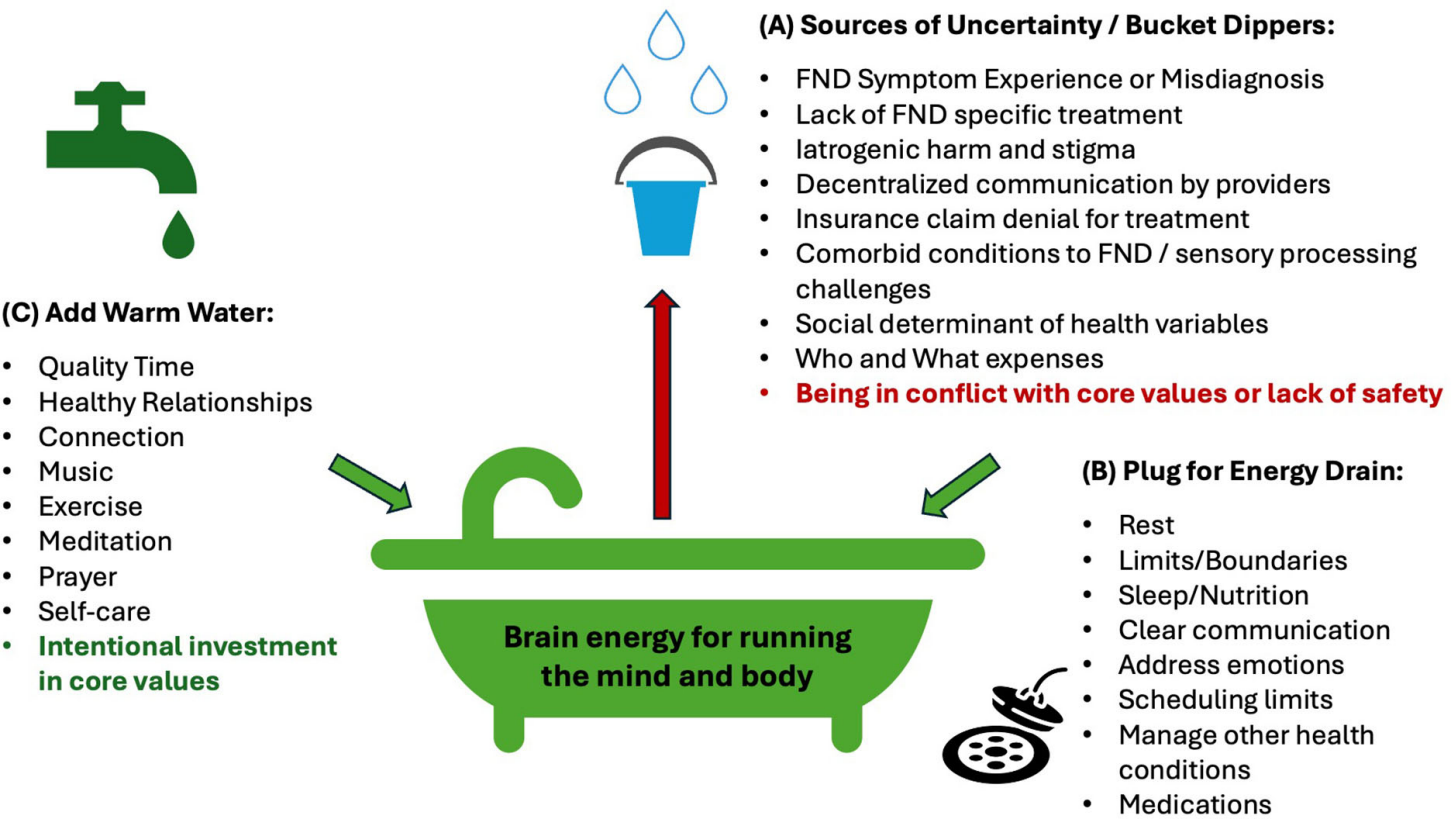
Treatment targets in FND. In **(A)** we highlight sources of uncertainty or “bucket dippers” that deplete the brain’s energy for running the mind and body. In **(B)** we highlight treatment strategies focused on self care and prioritizing expenses to the brain-body budget. In **(C)** we highlight treatment strategies focused on infusing vitality and joy back into life and the daily experience of the person with FND. We point out that FND treatment frequently focuses on **(B)**, and occasionally focuses on **(A)** which are both important, however, until **(A)** is properly addressed and uncertainty is reduced, no amount of progress with **(B)** and **(C)** will achieve sustained improvement of FND symptoms or prevent symptom migration.

In the context of the model proposed, with uncertainty as an operative construct, perpetuated by an environment contributing to high allostatic load, treatment for FND must provide a person with multiple explicit tools for confronting and reconciling uncertainty in the moment (**Figure 2**). The treatment approach must be tiered, first interrupting automatic patterns of high prediction and high activation so that then tools for updating sensory registrations and inferential models can have a solid and consistent foundation for future success. Such tools allow for accurate updating of sensory registrations and inferential models and will improve individuals’ ability to regulate a brain-body budget that balances allostatic challenge. It is our evidence-based belief that, first, the brain and nervous system need to be slowed so that certainty can be re-introduced in the body. Slowing interrupts the hyperactivation that leads to more prediction error and less energy to reconcile prediction error in FND. The first goal of proposed FND intervention is to convert an unconscious process of hyperactivation to a mindful and strategic process of activation and engagement. Slowing can modify “bottom-up” sensory processing, while also modifying “top-down” inferences about what sensory information means. Slowing the brain and body creates certainty using behavioral exercises to shift towards increased parasympathetic nervous system activation. This can be done with meditation, body scan practice, vagus nerve stimulation interventions such as the physiological sigh, cold plunges and learning to shift out of reactive states so that the brain has a chance to intentionally respond. Though, interventions utilizing these techniques have not targeted uncertainty but rather changes in psychiatric comorbidities (e.g., anxiety) or coping with stress.

Unlike the delivery offered in traditional CBT wherein such behavioral practices are framed as in service of relaxation, the frame of allowing for reintroduction to uncertainty is novel in our proposed approach, as a means of giving the person with FND immediate agency and top-down relief over symptoms. We propose that the initial stage of behavioral skills learning is necessary for establishing the foundation on which latter tools can follow for committed action to values, increased adaptive capacity, and increased agency over body and life. We believe that this foundation may be crucial for success with later interventions such as CBT, Acceptance and Commitment Therapy, and Dialectical Behavioral Therapy. This approach is consistent with evidence-based approaches encouraging emotional exposure (56). Tools for this model emphasize emotional acceptance and the ability to cultivate flexibility in responding to cognitive, emotional and physical experiences. Only until the individual with FND gains mastery of slowing and experiencing can they begin to consider what may be facilitating uncertainty and the symptoms (**Figure 2A**). For example, many current approaches to FND treatment focus on restoration (**Figure 2B**) or repletion of resources (**Figure 2C**), but until an individual masters tools for attenuating the impact of uncertainty, the functional neurological symptoms will persist. Many times, the foundation of symptom generation develops from higher-order psychological constructs such as value misalignment, experiential avoidance and other nuanced internal processes (4).

## Acceptance and commitment therapy

We believe that uncertainty is not just an association in FND, but rather, an operative construct in the formation and perpetuation of FND. Uncertainty, be it internal, external, physiological, social, or psychological, keeps the brain from being able to intentionally update sensory registration and/or from being able to intentionally update inferential models. Once a person is capable of intentional action, and has developed awareness from the contrast of automatic to intentional activations, the brain can get back to its homeostatic functions of running the body, and is no longer slowed by inevitable allostatic challenges in daily life. Although CBT and rehabilitation therapies such as PT/OT/SP have value in FND, it remains possible for FND symptoms to persist, or migrate into different phenotypes, until the person with FND has mastered tools for managing uncertainty. We therefore recommend that FND treatment targets recognize and address the operative role of uncertainty in FND symptom experience and continue to look for ways to reduce uncertainty for people having functional neurological symptoms.

A particularly compelling approach to intervene on intolerance of uncertainty and FND comes from “third wave” approaches including, Acceptance and Commitment Therapy (ACT). Whereas traditional CBT challenges cognitive distortions thereby inherently implying that the person’s thinking is wrong, ACT emphasizes consideration of the utility of thoughts while also cultivating psychological flexibility to emotional and physical experiences, including uncertainty. ACT emphasizes committed action based on values along with workability in regard to other aspects of experience. In this way, ACT may be more palatable than CBT to those with FND, as ACT does not challenge uncertainty, and makes no effort to convince the patient to directly change how they are thinking about their symptoms. Rather, ACT encourages them to change their response and relationship to their experience of symptoms and associated thoughts and emotions, providing a different avenue for intervention, and facilitating an experience of control. In ACT, the emphasis is on embracing uncertainty and the possibility of prediction error garnered via an attitude of willingness and focus on value-guided behavioral action in spite of challenging thoughts, emotions and physical experiences. This therapeutic approach does not see uncertainty or even IU as dysfunction, but rather as opportunities to optimize and build further adaptive capacity. When individuals can learn to intentionally accept experiences that arise, and put them in the context of their values, relief is the likely byproduct.

A few groups have begun studying ACT intervention in FND. In a study of eight patients with mixed manifestations of FND, five patients demonstrated reliable improvements in symptom interference/impairment after 6–8 sessions of intervention (56). More recently, feasibility of ACT intervention for functional cognitive disorder was documented in a cohort of 44 individuals randomized to ACT-specific versus treatment as usual intervention (57). The researchers documented that 64% of the ACT participants reported satisfaction with the intervention relative to 0% of the treatment as usual group. Other groups have additionally suggested the utility of ACT in FND treatment (57, 58). We believe ACT may be of great value in FND because it refocuses attention away from prediction error and interpretations of such errors, cognitively and emotionally, focusing instead of mindful acceptance of moment-to-moment experience, including uncertainty, so as to free body and brain resources to incrementally and optimally engage in value-consistent behavioral action. This approach focuses on allocating resources intentionally and strategically, using mindful awareness and acceptance tools to change the relationship between core self (“contextualized self” in ACT language), and unhelpful thoughts, while “making room” for challenging feelings, allowing for resetting of the relationship between prediction error and emotional catastrophizing.

## Dialectical behavior therapy and mindfulness

Dialectical behavior therapy (DBT) and mindfulness therapy are two additional modalities that offer concrete behavioral tools and strategies for improving distress tolerance and learning how to intentionally slow the brain and nervous system. The focus on the “Wise Mind” in DBT allows for the balance of rational and emotional factors in making decisions and behaving adaptively. DBT also emphasizes the importance of clear and direct communication, not just with oneself, but with others. For example, the “DEAR MAN” strategy teaches people how to enhance directness in communicating content (Describe, Express, Assert, Reinforce) but also how to approach direct communication in process (Mindful, Appear Confident, and Negotiate). This particularly strategy for making communication more direct diminishes uncertainty and associated interpersonal distress (59, 60). DBT tools can attenuate uncertainty within the individual with FND but also can attenuate uncertainty communicated between the individual with FND and others with whom they must interact. Any FND treatment must establish safety and trust between patient and medicine, allowing for the possibility to address and respond to all factors that reinforce continued uncertainty.

Using the proposed framework, we propose a tiered approach to FND treatment beginning with behavioral skills to reset the brain and nervous system, followed by ACT, DBT, and mindfulness tools that rewire and build up adaptive capacity, agency, and self-efficacy in life. The first stage of treatment should focus on conscious interruption of automatic patterns of brain network hyperactivation. The second stage of treatment should focus on learning explicit strategies to help people reduce the potential for uncertainty and the negative impact of uncertainty on their lives. In the second stage of intervention, skills are used to develop awareness of where uncertainty enters into mind and body function, and to develop tools for greater effectiveness in emotional regulation, communication, and allocation of energy and resources. This second stage of treatment is uniquely focused on building adaptive capacity for individuals with FND. Uncertainty is an operative construct in FND so building tolerance of uncertainty is an imperative step to intervening on FND symptoms. Future research can evaluate whether proposed interventions increase self-control, self-efficacy, and management of FND symptoms, demonstrating that a previously automatic and unconscious process can be made intentional, conscious, and strategic in building back health and wellness for people with FND.

## Conclusion

The emergent FND community is uniquely positioned in that it is simultaneously building its scientific, clinical and advocacy foundations. Unlike other neurological conditions that benefit from decades of research, established treatment protocols and supportive infrastructure, FND continues to lack a cohesive, recognized and respected system of care. At present, researchers are working to understand the brain mechanisms underlying FND, develop consensus guidelines, design and measure effective interventions and raise public and professional awareness. This contemporaneous development creates significant uncertainty for all members of the FND community. The uncertainty inherent to FND at all levels needs to be what is targeted in intervention and to optimize function. Otherwise, symptoms may persist and even migrate to new phenotype, with no change in disability, and no return to wellness and good health for individuals with FND. Limited confidence to define the neurological mechanisms that generate FND symptoms ends up limiting the ability to ascertain triggers and sustaining factors. Absent this information, FND interventions are merely experiments without promising direction, potentially resulting in superficial conclusions about operative factors, and further clouding certainty.
